# In-frame deletion variant of *ABCD1* in a sporadic case of adrenoleukodystrophy

**DOI:** 10.1038/s41439-025-00309-z

**Published:** 2025-02-28

**Authors:** Takashi Matsukawa, Atsushi Sudo, Toshiyuki Kakumoto, Akihito Hao, Mitsuhiro Kainaga, Hyangri Chang, Tatsuo Mano, Hiroyuki Ishiura, Jun Mitsui, Toshihiro Hayashi, Shinichi Morishita, Shoji Tsuji, Tatsushi Toda

**Affiliations:** 1 Department of Neurology, Graduate School of Medicine, The University of Tokyo, Bunkyo-ku, Japan; 2 Department of Neurology, Okayama University Graduate School of Medicine, Dentistry and Pharmaceutical Sciences, Okayama, Okayama, Japan; 3 Department of Precision Medicine Neurology, Graduate School of Medicine, The University of Tokyo, Bunkyo-ku, Japan; 4 Department of Computational Biology and Medical Sciences, Graduate School of Frontier Sciences, The University of Tokyo, Kashiwa, Japan; 5 Institute of Medical Genomics, International University of Health and Welfare, Narita, Japan

**Keywords:** Medical genetics, Mutation

## Abstract

Adrenoleukodystrophy (ALD), an X-linked leukodystrophy caused by pathogenic variants in *ABCD1*, exhibits a broad range of phenotypes from childhood-onset cerebral forms to adult-onset adrenomyeloneuropathy (AMN). We report a rare in-frame *ABCD1* deletion c.1469_71delTGG (p.Val490del) in a man with AMN. Although this variant has been interpreted as ‘uncertain significance’ in ClinVar, biochemical analysis along with clinical evaluation confirmed the pathogenicity of this variant, underscoring the importance of functional assessment of in-frame deletions.

ALD is an X-linked neurological disease caused by pathogenic variants of *ABCD1* (MIM #300371), which codes for ALD protein (ALDP), a peroxisomal membrane transporter directly involved in the import of very-long-chain fatty acids. Dysfunctions of ALDP lead to elevated levels of very-long-chain saturated fatty acids (VLCFAs) in the blood and tissues and the development of ALD. There is a wide variety of clinical phenotypes of ALD, including childhood-onset cerebral ALD, adult-onset cerebral ALD and AMN even in the same family^1,2^. In ALD, a wide variety of pathogenic variants have been reported, including missense, frameshift and structural variants^1,2^. Although pathogenic in-frame variants have also been identified in some patients^1,2^, the interpretation of the pathogenicity of in-frame (protein-length-changing) variants is challenging, because the functional outcomes may be variable. Here, we describe a case of a patient with AMN harboring an in-frame deletion, for which we experienced difficulties in interpreting the pathogenicity of an in-frame deletion.

The patient first developed spasticity of the lower limbs at the age of 37 years, which gradually progressed since then. There was no family history of any neurological diseases. At the age of 42, he noticed a delay in initiating urination and a sensation of incomplete bladder emptying. At the age of 51, an evaluation for frequent urination was conducted, and a neurogenic bladder due to storage dysfunction was diagnosed. At the age of 60, brain magnetic resonance imaging (MRI) showed mild cerebral and cerebellar atrophy, as well as small white-matter lesions in the periventricular, frontal lobe and brainstem regions, where chronic ischemic changes could not be excluded (Fig. 1A). The clinical presentation of this patient is considered spastic paraplegia, a form of neurodegenerative disease. Because spastic paraplegia is a group of heterogeneous diseases, the establishment of an accurate diagnosis is often difficult.

**Fig. 1:**
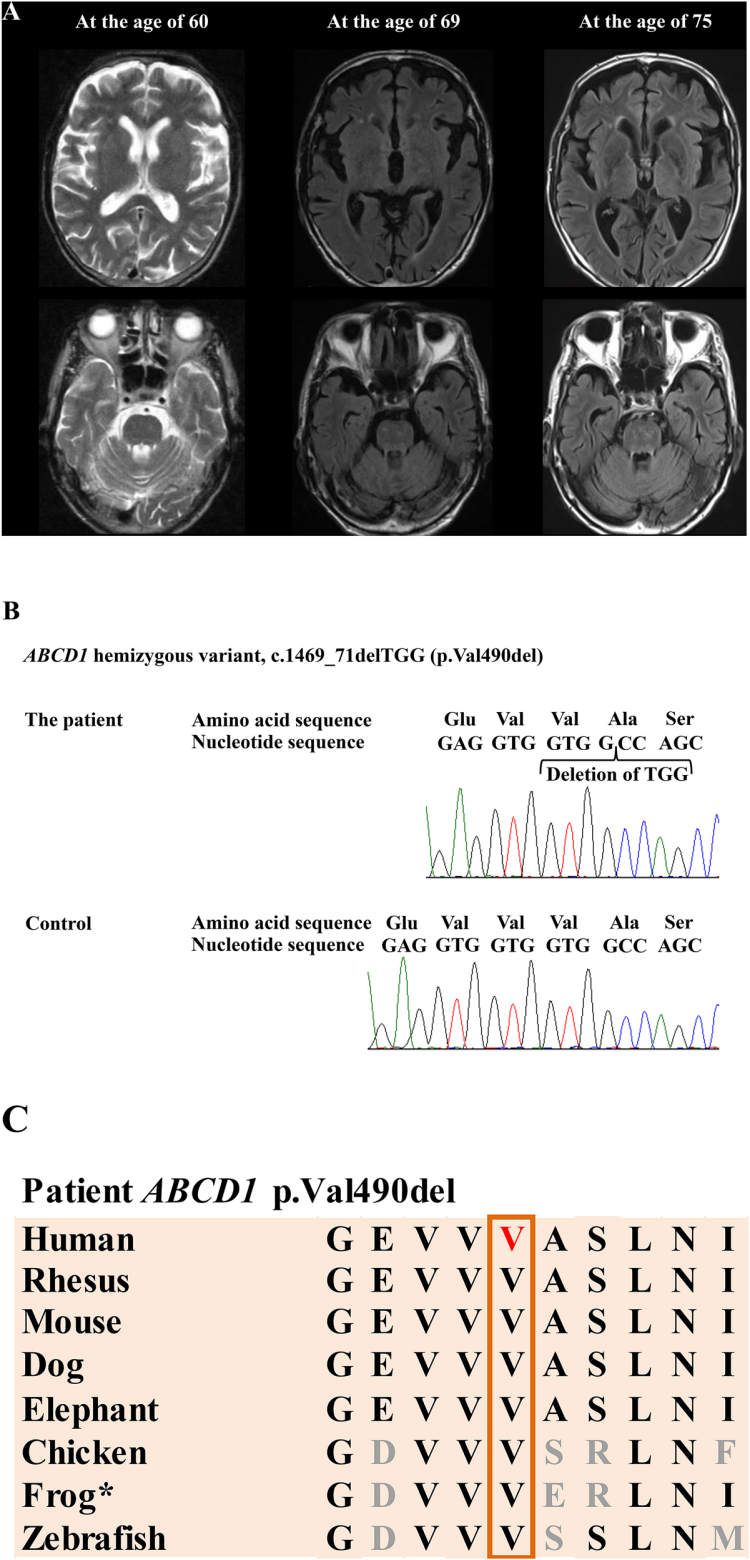
Changes in brain magnetic resonance (MR) images and results of genetic analysis of *ABCD1.* **A** Brain MRI findings. Left: white-matter lesions of the periventricular, frontal lobe and brainstem regions, and the mild atrophy of the cerebrum and cerebellum on the axial T2-weighted brain MR images at the age of 60. Middle: white-matter lesions of the periventricular, frontal lobe and brainstem regions, and the mild atrophy of the cerebrum and cerebellum on the axial fluid-attenuated inversion recovery (FLAIR) brain MR image at the age of 69. Right: white-matter lesions of the periventricular, the frontal lobe and brainstem regions, and the mild atrophy of the cerebral and cerebellum on FLAIR brain MR image at the age of 75. Since the age of 60, there have been no significant changes in the white-matter lesions. The atrophic changes in the cerebrum and cerebellum progressed slightly. **B** Direct nucleotide sequence analysis of the region where the variant was detected. The hemizygous variant c.1469_71delTGG (p.Val490del) in *ABCD1* was identified. **C** Comparison of amino-acid sequences around the variant p.Val490del in *ABCD1* among species. The amino-acid sequences around the variant p.Val490del in *ABCD1* were fairly preserved among species up to zebrafish. *, *Xenopus tropicalis*.

At the age of 69, neurological examination revealed bilateral pyramidal signs, pyramidal weakness of the lower limbs, left-dominant mild ataxia of the upper limbs, decreased vibration sensation in the lower limbs, right-side-predominant hearing impairment and bladder dysfunction. His Mini-Mental State Examination Japanese version (MMSE-J) score was 30 out of 30, and brain MRI showed slight progression of brain atrophy (Fig. 1A). Whole-exome sequencing was performed, and the in-frame deletion variant c.1469_71delTGG (p.Val490del) in *ABCD1* (NM_000033.4) was identified. This variant was confirmed by direct nucleotide sequence analysis of polymerase chain reaction products using specific primers for exon 6 (Fig. 1B and Supplementary Table 1)^2^. Further nucleotide sequence analysis of all the exons and the exon–intron boundaries of *ABCD1* with specific primers did not reveal any other variants except for c.1469_71delTGG (p.Val490del).

The frequency of this variant in the population database in Japan (ToMMo 60KJPN dataset, https://jmorp.megabank.tohoku.ac.jp/) is 0.000011 (1 allele in one control male subject among 98,731 alleles). This variant is not registered in gnomAD v4.1.0 (https://gnomad.broadinstitute.org/). The in-frame deletion is present within the ATP-binding region of ALDP, a region where other known causative variants have been reported. Evolutionary conservation of the affected amino acid, valine at position 490, is observed across species, from humans through zebrafish, as shown in the UCSC Genome Browser (https://genome.ucsc.edu/) (Fig. 1C). The Combined Annotation Dependent Depletion (CADD) PHRED score of this variant was 18.04, supporting its damaging effect^3^. Furthermore, SpliceAI analysis did not suggest any evidence of acceptor loss, donor loss, acceptor gain or donor gain, with delta scores of 0.04, 0.01, 0.00 and 0.00, respectively^4^. According to the American College of Medical Genetics and Genomics (ACMG) guidelines^5^, the variant fulfills only PM4 (a protein-length-changing variant) and PP3 (multiple lines of computational evidence support a deleterious effect on the gene or gene product), indicating weak evidence for the pathogenicity. Thus, it is difficult to conclude that the variant is pathogenic for ALD on the basis of the ACMG guidelines.

In the ALD variant database (https://adrenoleukodystrophy.info/mutations-and-variants-in-abcd1), this variant is listed as a likely deleterious variant, although clinical and familial information remains unpublished, whereas in the Human Gene Mutation Database (https://www.hgmd.cf.ac.uk/ac/index.php), this variant is also recorded without any published clinical and familial information (CD107825). The variant is registered as a variant of ‘uncertain significance’ (RCV001002582.8 and RCV003490006.2) in ClinVar (https://www.ncbi.nlm.nih.gov/clinvar/) with the following comment: this variant deletes a single valine residue, leaving the rest of the protein in-frame. However, given the lack of clinical and functional data, the significance of the p.Val490del variant is uncertain at this time.

Biochemical testing showed elevated levels of VLCFAs in plasma sphingomyelin (C24:0/C22:0: 1.738 (reference range 0.628–0.977), C25:0/C22:0: 0.059 (0.012–0.023), C26:0/C22:0: 0.026 (0.003–0.006)), which confirmed the diagnosis of ALD. From the viewpoint of the ACMG guidelines, the results of biochemical analysis support PS3 (well-established functional studies show a deleterious effect), leading to the classification of the variant as ‘likely pathogenic’ (PS3, PM4 and PP3).

The phenotype of the patient is consistent with AMN, as his main symptoms included gradually progressive spasticity of the lower limbs and bladder dysfunction and brain MRI revealed only small white-matter lesions where chronic ischemic changes could not be excluded, although neurological symptoms also indicated slight limb ataxia and brain MRI showed mild cerebral and cerebellar atrophy. At the age of 74, his MMSE-J score was 26 out of 30 (digit span backward −3, recall −1). However, his brain MRI did not show the appearance of new white-matter lesions (Fig. 1A). At the age of 76, he was diagnosed with middle pharyngeal cancer at stage IVB and received the best supportive care. He died at the age of 76 owing to the progression of middle pharyngeal cancer and the resulting aspiration pneumonia.

As described above, we should point out issues in the interpretation of rare in-frame (protein-length-changing) variants. As indicated by the ACMG guidelines, in-frame variants per se are classified as PM4, which alone does not fulfill the criteria for pathogenicity. Furthermore, this is not a novel variant, with 1 allele registered among 98,731 alleles. Given the increasing number of participants in the population database, extremely rare variants that are pathogenic for diseases may be registered especially for late-onset diseases. Thus, the classification of PM2, which is absent from population databases, as a novel variant needs to be reconsidered. Furthermore, it is not always possible to perform functional studies for every variant identified in genetic testing. Our findings further highlight the importance of enriching disease-specific variant databases with rich clinical information, information regarding the interpretation of the pathogenicity of variants and, if available, the results of functional studies, which should help in evaluating the pathogenicity of variants that may remain variants of uncertain significance.

## HGV Database

The relevant data from this Data Report are hosted at the Human Genome Variation Database at 10.6084/m9.figshare.hgv.3482.

## Supplementary information

Supplementary Table 1
