# Supplementary material for: In-frame deletion variant of ABCD1 in a sporadic case of adrenoleukodystrophy
Source: Hum Genome Var. 2025 Feb 28;12:5. doi: 10.1038/s41439-025-00309-z (PMC11871001; doi:10.1038/s41439-025-00309-z)
Supplement: Supplementary file 1 — Supplementary Table 1 [file 41439_2025_309_MOESM1_ESM.docx]

Supplementary Table 1

*ABCD1*PCR primer pairs

| Exon | Primer | Primer length (bp) | Amplicon size (bp) |
| --- | --- | --- | --- |
| Exon 1F | 5’-TGACAGGACAGGAGAGCCAAGTTC | 24 | 1547 |
| Exon 1R | 5’- AACCGCTAGGATCGCAGCTCTAAG | 24 |  |
| Exon 2F | 5’- TACACCTTGAGTTTGAGACCTGGC | 24 | 640 |
| Exon 2R | 5’- AGGGTATCTGTGCCTGGAGAAGTG | 24 |  |
| Exon 3F | 5’- CTGGGTTGGTTTGTCTGTATGGTG | 24 | 847 |
| Exon 4R | 5’- GCAGCTACTGTCTGGGAAGGAAGG | 24 |  |
| Exon 5F | 5’- GGAAGAGTTCAGCTTGTTGGAAGACC | 26 | 438 |
| Exon 5R | 5’- TCAGAGACACGTTGGTCTCTCACC | 24 |  |
| Exon 6F | 5’- CTACTCATTCAGCTGTGGCAGAATAGG | 27 | 2181 |
| Exon 7R | 5’- AGGCCAAACACAGCAGAGTGACC | 23 |  |
| Exon 8F | 5’- CCATAAACCGCAGGGATGGATTG | 23 | 1636 |
| Exon 10R | 5’- GACTCGAGTCTCTGGAGGGAGG | 22 |  |

*ABCD1* Sanger sequencing primers

| Exon | Primer | Primer length (bp) |
| --- | --- | --- |
| Exon 1dF1 | 5’- TCAACTGCTGCCCCAGG | 17 |
| Exon 1dF2 | 5’- GTCCTGTGCCGGGAGAC | 17 |
| Exon 1dF3 | 5’- TCCCAGCAGACCTACTACCG | 20 |
| Exon 1dR1 | 5’- CCAGCCTTCCGTCCAGG | 17 |
| Exon 1dR2 | 5’- CAGGTTGGAGTAGAGGTGGG | 20 |
| Exon 1dR3 | 5’- GACTGTCCCCACCGCTC | 17 |
| Exon 2F | 5’- TACACCTTGAGTTTGAGACCTGGC | 24 |
| Exon 2R | 5’- AGGGTATCTGTGCCTGGAGAAGTG | 24 |
| Exon 3dF1 | 5’- GGCACCATTTGCAGAAGAG | 19 |
| Exon 3dR1 | 5’- CCTGCCAGTTGCAGGGAGAG | 20 |
| Exon 4dF1 | 5’- CATCCTTGCCATGCTTCTC | 19 |
| Exon 4R | 5’- GCAGCTACTGTCTGGGAAGGAAGG | 24 |
| Exon 5dF1 | 5’- CTGCCAGGGATGGGAATGAG | 20 |
| Exon 5R | 5’- TCAGAGACACGTTGGTCTCTCACC | 24 |
| Exon 6dF1 | 5’- CATAGGGTACGGGAAGGGG | 19 |
| Exon 6dR2 | 5’- GCTCTGGGGACAGTGCCTC | 19 |
| Exon 7dF1 | 5’- GGAGGCGCAGAGTATCTTGG | 20 |
| Exon 7dR1 | 5’- TGTTGAGGTAATCTTCACTCCCTG | 24 |
| Exon 8dF1 | 5’- CCTGTCGTCACAGCTAGCTC | 20 |
| Exon 8dR1 | 5’- CAGGCTCCACTGAGCCCAG | 19 |
| Exon 9dF1 | 5’- GGGCTCAGTGGAGCCTGAG | 19 |
| Exon 9dR1 | 5’- AGGCCACCTCCTCCCCTCAG | 20 |
| Exon 10dF1 | 5’- CTGTGGTAGGTGCCCTGTC | 19 |
| Exon 10dR1 | 5’- GTCTCCTTCATGTGATCCGAGC | 22 |
